# Supplementary material for: Impact of hfq and sigE on the tolerance of Zymomonas mobilis ZM4 to furfural and acetic acid stresses
Source: PLoS One. 2020 Oct 9;15(10):e0240330. doi: 10.1371/journal.pone.0240330 (PMC7546472; doi:10.1371/journal.pone.0240330)
Supplement: S1 Table — (DOCX) [file pone.0240330.s001.docx]

**Impact of *hfq* and *rpoE* on the tolerance of *Zymomonas mobilis* ZM4 to furfural and acetic acid stresses**

S1 Table: Primers used for the preparation of recombinant plasmids and qPCR

| Primer | Sequence (5´–3´) | Restriction  site underlined | Gene to be amplified |
| --- | --- | --- | --- |
| pdc-F | TAATCACTTAATCCAGAAACGGG |  | Promoter of pyruvate decarboxylase |
| pdc-R | TGCTTACTCCATATATTCAAAACAC |  | Promoter of pyruvate decarboxylase |
| Tpdc-F | TAGTTTTTAAATAAACTTAGAG |  | Terminator of pyruvate decarboxylase |
| Tpdc-R | AATTTTATAGAAAAGAAAAACAAAG |  | Terminator of pyruvate decarboxylase |
| sig-F | ATGGAAAATCATGAAAAAGAGA |  | ZMO1404 |
| sig-R | CTAGCGTCCGTTACTAAGG |  |  |
| hfq-F | ATGGCAGAAAAGGTCAACAAT |  | ZMO0347 |
| hfq-R | TCAATCCTCGTCTCGCCT |  |  |
| pdc-F´ | CGCGGATCCTAATCACTTAATCCAGAA | *Bam*HI |  |
| pdc-R-sig | CATGATTTTCGATCATTGCTTACTCCATATATTC |  |  |
| pdc-R-hfq | GTTGACCTTTTCTGCCATTGCTTACTCCATATATTC |  |  |
| sig-F-pdc | GAATATATGGAGTAAGCAATGATCGAAAATCATG |  |  |
| sig-R´ | CCGGAATTCCTAGCGTCCGTTACTAAG | *Eco*RI |  |
| hfq-F-pdc | GAATATATGGAGTAAGCAATGGCAGAAAAGGTCAAC |  |  |
| hfq-R´ | CCGGAATTCTCAATCCTCGTCTCGCCT | *Eco*RI |  |
| pdc-F-q | TACAACCTCGTCCTTCTT |  | ZMO1360 |
| pdc-R-q | CATAACCTTCTGCACTGA |  |  |
| adh-F-q | GGTATTAATTCTGCTGTT |  | ZMO1596 |
| adh-R-q | CGAAGTCTGAATTGTTAT |  |  |
| sig-F-q | AGGATTCTTCTCCAATTACTG |  |  |
| sig-R-q | GTCACGATTGCCCGAA |  |  |
| hfq-F-q | TAATACCTTGCGCAAGAC |  |  |
| hfq-R-q | TGGTAGAAATAGCGTGTTT |  |  |
| xylR-F-q | TTTACAGGCGATGATTTACG |  | ZMO0976 |
| xylR-R-q | AAGCCAACACCGATTTATT |  |  |
| *nha*A-F-q | TGTGATGGTATCAAAAGCGGTC |  | ZMO0117 |
| *nha*A-R-q | CCAAATCGGTGACACGGAA |  |  |
