# Supplementary material for: Impact of hfq and sigE on the tolerance of Zymomonas mobilis ZM4 to furfural and acetic acid stresses
Source: PLoS One. 2020 Oct 9;15(10):e0240330. doi: 10.1371/journal.pone.0240330 (PMC7546472; doi:10.1371/journal.pone.0240330)
Supplement: S2 Table — (DOCX) [file pone.0240330.s002.docx]

**Impact of *hfq* and *rpoE* on the tolerance of *Zymomonas mobilis* ZM4 to furfural and acetic acid stresses**

S2 Table: Some of the sugarcane bagasse hydrolysate ingredients

| Component | Concentration (gL^-1^) |
| --- | --- |
| Glucose | 11.56 |
| Furfural | 1.32 |
| Acetic acid | 3.61 |
| 5-(Hydroxymethyl)furfural (HMF) | 0.08 |
| Total phenolic compounds | 0.78 |
