# Supplementary figures and images for: Impact of hfq and sigE on the tolerance of Zymomonas mobilis ZM4 to furfural and acetic acid stresses
Source: PLoS One. 2020 Oct 9;15(10):e0240330. doi: 10.1371/journal.pone.0240330 (PMC7546472; doi:10.1371/journal.pone.0240330)

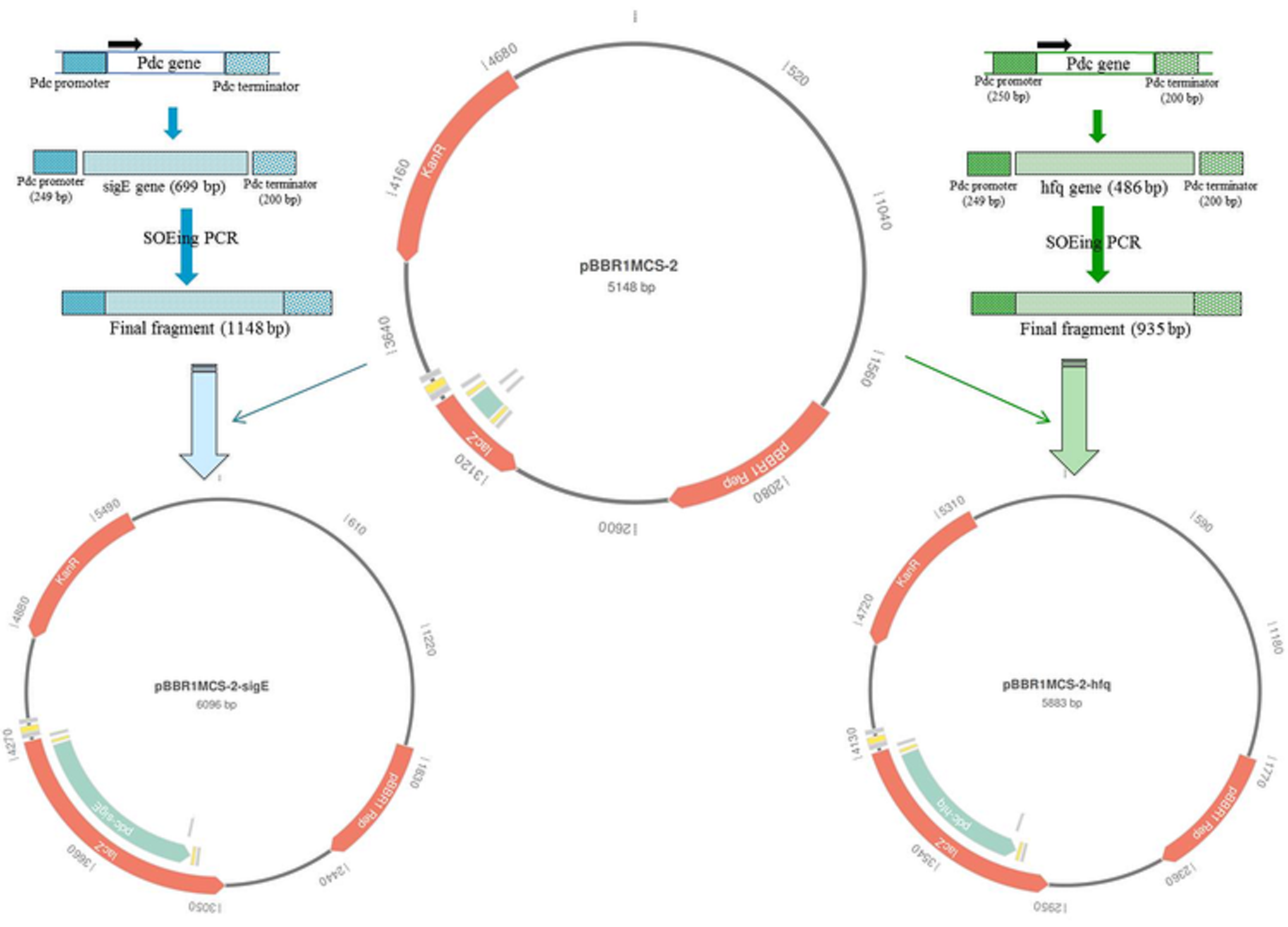

Supplement: S1 Fig — (TIF) [file pone.0240330.s003.tif]

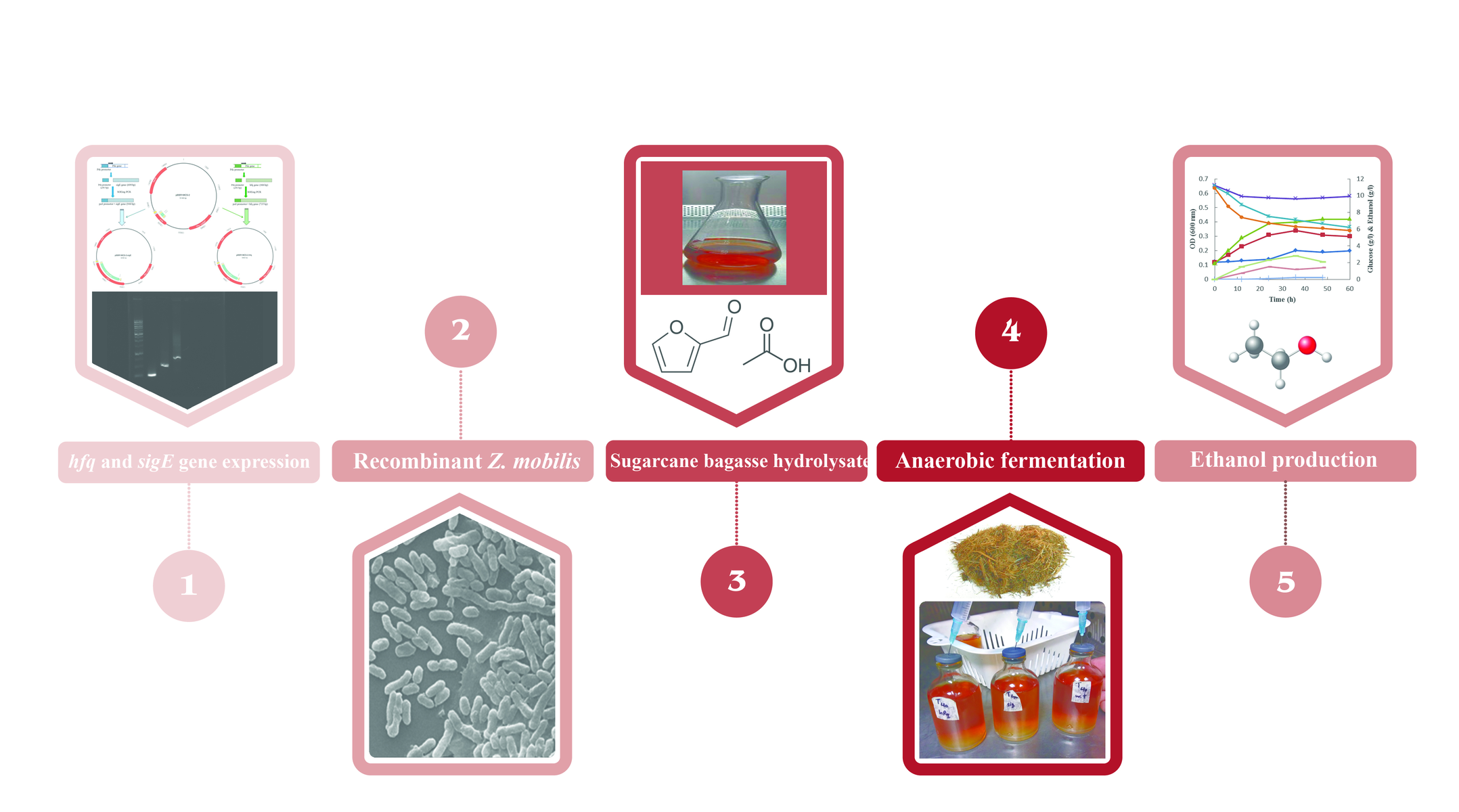

Supplement: S1 Graphical abstract — (TIF) [file pone.0240330.s004.tif]
