# Supplementary material for: Impact of hfq and sigE on the tolerance of Zymomonas mobilis ZM4 to furfural and acetic acid stresses
Source: PLoS One. 2020 Oct 9;15(10):e0240330. doi: 10.1371/journal.pone.0240330 (PMC7546472; doi:10.1371/journal.pone.0240330)
Supplement: Attachment — Submitted filename: Response to Reviewers.docx [file pone.0240330.s005.docx]

30 July 2020

Dear Prof. Heber

# Editor in Chief of PLOS ONE

We are very excited to have been allowed to revise our manuscript "Impact of *hfq* and *sig*E on the tolerance of *Zymomonas mobilis* ZM4 to furfural and acetic acid stresses" (Manuscript Number: PONE-D-20-14531). Thanks for the advice, which will help us improve it to a better scientific level. We have revised the manuscript according to the editor's report and reviewer's comments and the proposed changes have taken place. We believe that the manuscript is improved after making the suggested editions and we hope our revisions meet your approval.

Yours Sincerely

Hamid Moghimi

Email: [hmoghimi@ut.ac.ir](mailto:hmoghimi@ut.ac.ir)

Journal Requirements:

When submitting your revision, we need you to address these additional requirements.

1. Please ensure that your manuscript meets PLOS ONE's style requirements, including those for file naming. The PLOS ONE style templates can be found at

  -Thanks to the editor for the comment, the manuscript format has been corrected according to PLOS ONE's style requirements.

2. Thank you for stating the following in the Acknowledgments Section of your manuscript:

"The University of Tehran partially supported this work." We note that you have provided funding information that is not currently declared in your Funding Statement. However, funding information should not appear in the Acknowledgments section or other areas of your manuscript. We will only publish funding information present in the Funding Statement section of the online submission form.

Please remove any funding-related text from the manuscript and let us know how you would like to update your Funding Statement. Currently, your Funding Statement reads as follows: "The author(s) received no specific funding for this work."

  -Thanks to the editor for the comment, Acknowledgments Section was edited.

3. Please ensure that you refer to "Figure Graphical Abstract" in your text as, if accepted, production will need this reference to link the reader to the figure.

 -Thanks to the editor for the comment, Graphical Abstract has been cited in the manuscript (line 115-117)

4. Please include captions for your Supporting Information files at the end of your manuscript, and update any in-text citations to match accordingly. Please see our Supporting Information guidelines for more information: [http://journals.plos.org/plosone/s/supporting-information](about:blank).

-Supporting information legends were added at the end of the manuscript and correctly cited in the text.

5. Review Comments to the Author

Please use the space provided to explain your answers to the questions above. You may also include additional comments for the author, including concerns about dual publication, research ethics, or publication ethics. (Please upload your review as an attachment if it exceeds 20,000 characters)
Reviewer #1: Comments to PONE-D-20-14531

The authors constructed two stress tolerant *Zymomonas mobilis* strains by introducing hfq and sigmaE. The topic sounds interesting. However, the research lacks innovation.

Q1: Actually, Yang *et al* has used this approach to construct stress tolerant strain by overexpression hfq in *Z. mobilis* (Shihui Yang, Dale A Pelletier,Tse-Yuan S Lu, and Steven D Brown. The *Zymomonas mobilis* regulator hfq contributes to tolerance against multiple lignocellulosic pretreatment inhibitors. BMC Microbiol, 2010; 10: 135). In another research, Tiago *et al* also used different sigma factor to construct stress tolerant *Z. mobilis* strains (Tiago Benoliel, Marciano Régis Rubini, Carolina de Souza Baptistello, Christiane Ribeiro Janner, Vanessa Rodrigues Vieira, Fernando Araripe Torres, Adrian Walmsley & Lidia Maria Pepe de Moraes. Physiological effects of overexpressed sigma factors on fermentative stress response of *Zymomonas mobilis*. Brazilian Journal of Microbiology, 2020, 51: 65-75)

A1: In the study of Yang *et al*. developed a *Z. mobilis hfq* insertion mutant. The resulting strain designated as AcRIM0347 and its role was investigated in model lignocellulosic inhibitors including acetate, vanillin, furfural and hydroxymethylfurfural. Also, they named pBBR3DEST42 for *hfq* expression (p42-0347). According to their results, only *Z. mobilis hfq* mutant (AcRIM0347) was assayed in the presence of model lignocellulosic pretreatment inhibitors. In our study, the overexpression of *hfq* in ZM4 strain and its behaviour in furfural, acetic acid and multi-complex inhibitors was investigated. Besides, in the Yang study, there is no evidence about ethanol production and their focus was mainly on the growth of *Z mobilis*.

Moreover, the recombinant *hfq* expression profile in the presence of multi-complex of lignocellulosic inhibitors was not analyzed. In our study, for the first time, the relation between sugar consumption, ethanol and biomass production were investigated in wild type and recombinant *hfq* *Z. mobilis* in the high and low concentrations of lignocellulosic inhibitors. Finally, the behaviour of recombinant strain was studied in real sugarcane hydrolysate with complex inhibitors and the expression level of some indicator genes were compared in recombinant strain. We believe our study affords more insight about the *hfq* as a multifunctional global regulatory factor.

In the study of Benoliel *et al*. they overexpressed sigma 32 and sigma 24 (Sig E) in *Z. mobilis* and their influence were tested in response to osmotic and ethanol stresses. Here, we studied the overexpression of sigma 24 (Sig E) in response to the lignocellulosic inhibitors stresses for the first time. According to Benoliel *et al*. results, it was not possible to conclude if sigma 24 affects ethanol tolerance in *Z. mobilis*, but the overexpression of this sigma factor led to a decrease in ethanol productivity. To our knowledge, our study is the first report of Sig E overexpression in *Z. mobilis* that has evaluated the recombinant strains on the lignocellulosic inhibitors such as furfural and acetic acid. Based on our results, in comparison to Benoliel *et al*. study, Sig E overproduction has significant effects on cell tolerance to the lignocellulosic inhibitors and improves ethanol production in comparison to wild type strain.

We believed that our detailed experimental achievements about two transcriptional elements help to gain deep insights in *Z. mobilis* stress response mechanisms.

Respect to the reviewers' comment, the novelty of the study was more highlighted in the manuscript (line 86-96).

Reviewer #2: This study evaluated the effect of two transcriptional elements, hfq and sigE, on stress tolerance of *Z. mobilis* to furfural, acetic acid and sugarcane hydrolysate. It demonstrated the role of hfq and sigE on resistance to toxic byproducts from pretreatment. Although this manuscript presented a complete study, there are many concerns should be addressed.

Thanks to the honorable reviewer of the article

Q1. The authors need to discuss and dig deeper in the results section rather than simply listing the trends of the diagram without extended analysis. There are a lot of interesting things to talk about, for example, in Table 3, why the expression of xylR and nhaA was significantly different between ZM4-hfq and ZM4-sigE. What are the potential regulation of hfq and sigE on expression of xylR or nhaA?

A1. We thank the reviewer for his/her constructive comment. Accordingly, to improve our discussion, we added the following paragraphs to the manuscript (lines 233-239, 245-249, 263-269, 271-273, 285-289, 299-301, 306-313, 331-336, 365-371, 410-419).

Q2. Line 23-25, please rewrite these sentences to ensure that they are rational.

A2. The mentioned sentences rewrote to clarify the meaning (lines 32-34).

Q3. Line 27-29, please revise this sentence.

A3. The mentioned lines revised (lines 35-38).

Q4. Line 49-51, provide references.

A4. References were added to the reference list.

Q5. Line 69, please reform the reference.

A5. The mentioned sentence was checked.

Q6. Line 144, please replace five with 5.

A6. A replacement was made.

Q7. Line 193-194, please make sure that this sentence is correct.

A7. The mentioned sentence was checked.

Q8. Figure 3 and Figure 4. Please adjust the color of line and shape of dots to make them clear.

A8. Based on the reviewer's comment, some changes were made in figures.

Q9. Figure 3a. Please explain why the glucose will slightly increase after reducing to 0 g/L.

A9. Prolonged starvation and nutrient deprivation will result in a drastic decline in optical density followed by cell death. As a consequence of the cell lysis, cellular contents as nutrients are released that is attributed to a slight increase in glucose content. The rest of the population scavenge these nutrients as carbon and nitrogen sources.

- Juana María Navarro Llorens, Antonio Tormo, Esteban Martínez-García, "Stationary phase in gram-negative bacteria", *FEMS Microbiology Reviews*, Volume 34, Issue 4, July 2010, Pages 476–495.
- Jaishankar, Jananee, and Preeti Srivastava. "Molecular basis of stationary phase survival and applications." *Frontiers in microbiology* 8 (2017): 2000.

Q10. Table 3, the author wrote "Avg±std" in the title, but no such format number was showed in this table.

A10. Thank the reviewer for precise comment, standard deviations for all quantifications were added in the manuscript.

Q11. The 1st and 2nd paragraph in "3. Results and Discussion" should be put into "1. Introduction".

A11. The mentioned paragraphs re-organized and put into the introduction section.

Q12. Figure 1 should be in supplementary materials

A12. According to the reviewer's comment, a replacement was done.

Q13. Table S3 should be in the main text. Because the authors cited it many times. It is important.

A13. Table S3 was added to the main text of the manuscript.

Q14. The language and sentence should be polished carefully.

A14. We thank the reviewer for this comment. A native speaker edited the manuscript.
